# Antimicrobial and antiproliferative activity of *Athamanta sicula* L. (Apiaceae)

**DOI:** 10.4103/0973-1296.75893

**Published:** 2011

**Authors:** Vita Di Stefano, Rosa Pitonzo, Domenico Schillaci

**Affiliations:** *Department of Chemistry and Pharmaceutical Technology, Faculty of Pharmacy, University of Palermo, Via Archirafi 32, 90123 Palermo, Italy*

**Keywords:** Antimicrobial activity, antiproliferative activity, *Athamanta sicula*, apiol, myristicin

## Abstract

**Background:**

*Athamanta sicula* L., a member of Apiaceae, is an annual perennial herb and it is known in Sicilian popular medicine with the name of “spaccapietre” (rock splitters), because fresh roots infusions are indicated as diuretic and used in the treatment of diseases of the urinary tract, and to dissolve kidney stones.

**Materials and Methods:**

Acetone extracts of leaves, flowers, and stems of *A. sicula* L. were investigated in vitro for antibacterial and cytotoxic activities. Antimicrobial activity was carried out against bacterial and fungal strains and antiproliferative activity against a group of human cancer cell lines (K-562, NCI-H460, and MCF-7).

**Results:**

All acetone extracts, apiol and myristicin, resulted inactive as antimicrobial agents at the maximum tested concentration of 200 μg/mL, but they induced significant antiproliferative activity on the tested cancer cell lines.

**Conclusions:**

Our study show that both apiol and myristicin could be tested as novel treatment in cancer chemotherapy.

## INTRODUCTION

It is noteworthy that Apiaceae species contain compounds with several and different biological activities, such as antibacterial, anticancer, hepatoprotective, vasorelaxant, and cyclooxygenase inhibition.[1–6]

*Athamanta sicula* L., a member of Apiaceae, is an annual perennial herb, 30-100 cm tall. It occurs in calcareous vertical cliffs at altitudes of 100-1500 m and is widespread in South Italy, particularly in Sicily. The habitat and ecologic and phytosociologic characteristics of the plant have been studied.[7–10]

*A. sicula* L. is known in Sicilian popular medicine with the name of “spaccapietre” (rock splitters), because fresh roots infusions are indicated as diuretic and used in the treatment of diseases of the urinary tract, and to dissolve kidney stones.[11–13] Previous data reported that the main constituent in A. sicula essential oil is apiol.[14,15]

This article presents the investigation of chemical constituents and antimicrobial and cytotoxic activities of acetonic extracts of aerial parts of the plant.

## Materials and Methods

### Plant material

Plant samples were collected in the flowering stage in Monte Pellegrino (606 m above sea level) near Palermo, in May 2006. A voucher specimen has been deposited in the Herbarium of Botanical Garden of Palermo (Italy).

### Extraction, purification, and isolation of chemical constituents

Aerial parts (leaves, flowers, and stems) of the plant were air dried, pulverized, and extracted (100 g for each part of the plant in 1 L of acetone) at room temperature for 24 h.

The extracts of each of the different parts of *A. sicula*, after evaporation of the solvent under reduced pressure, gave 3 semi-solid residues; 1.54 g from the leaves, 3.72 g from the flowers, and 0.746 g from the stems.

The dried extracts were submitted to flash chromatography on silica gel (Merck, Darmstadt, Germany, silica gel 60, 230-400 mesh), using petroleum ether-ethyl acetate (95:5). Further sequential purification by preparative thin layer chromatography using petroleum ether-ethyl acetate (98:2) yielded as chromatographically pure solids apiol (0.0016%) and myristicin (0.00002%) from leaves extract and only apiol from flower and stem extracts (0.043% and 0,0062%, respectively).

### Antimicrobial and antiproliferative assay

The antimicrobial activity of acetone extracts, apiol and myristicin, against reference strains American Type Culture Collection (ATCC) and German Collection of microorganism (DSM), *Staphylococcus aureus* ATCC 29213, *Staphylococcus epidermidis* DSM 3269, *Escherichia coli* ATCC 25922, *Pseudomonas aeruginosa* ATCC 9027, *Candida albicans* ATCC 10231, and *Candida tropicalis* ATCC 13803 was assessed. Testing media used were Müller–Hinton broth for bacteria and Sabouraud broth for fungi.

Appropriate volumes of samples of the 3 extracts and of apiol and myristicin (10 mg/mL solution in dimethyl sulfoxide [DMSO]) were added separately to 5 mL of broth in a series of test tubes so as to obtain a screening concentration of 200 µg/mL. Each set of the prepared samples was inoculated with 100 μL of bacterial suspension containing ≅10^6^ cfu/mL. The same number of samples was inoculated with 100 μL of a fungal suspension containing ≅10^5^cfu/mL. All of the samples were incubated at 37°C for 24 h.

In the same way, acetone extracts, apiol and myristicin, were tested *in vitro* for their antiproliferative activity against K-562 (human chronic myelogenous leukemia), NCI-H460 (human lung tumor), and MCF-7 (human breast adenocarcinoma) cell lines. The above-mentioned cell lines were grown at 37°C in a humidified atmosphere containing 5% CO _2_, in RPMI-1640 medium for K-562 or MEM (Sigma-Aldrich Corp., St. Louis, USA) in the case of MCF-7 and NCI-H460, supplemented with 10% fetal calf serum and antibiotics. K-562 cells were suspended at a density of 1 × 10 ^5^ cells/mL in growth medium, transferred to 24-well plate (1 mL per well) and incubated at 37°C for 48 h in the presence and in the absence (in the case of control wells) of a screening concentration of 100 μg/mL of the substance.

The viable cell number was calculated in a hemocytometer by dye exclusion with trypan blue 0.2% (w/v).[16]

The antiproliferative activity against NCI-H460 and MCF-7 was determined by methyltetrazolium (MTT) assay.[17] MTT is a yellow tetrazolium salt that is taken up and cleaved only by metabolically active cells, which reduce it to a colored, water-insoluble formazan salt. The solubilized formazan product can be quantified via absorbance at 570 nm (690 nm for blank), which is measured using a 96-well format spectrophotometer. The absorbance correlates directly with the cell number. The cells were plated at 2.0 × 10^4^ cell/well in 100 μL volume in 96-well plates and grown for 72 h in MEM complete medium. Different concentrations of test drugs or 0.1% DMSO were added to the wells. Then the cells were incubated with 10 μ of MTT (5 mg/mL) at 37°C for 3 h. The tetrazolium crystals were solubilized by the addition of 4.5 mL of isopropyl alcohol and 0.5 mL of Triton X-100 in 150 μL of 37% HCl.

From the dose-response curves, IC _50_ values (concentrations that induce 50% inhibition of cell growth) were calculated.

## Results

### Characterization of compounds

The structures of the compounds were elucidated by physicochemical and spectral analysis and were in agreement with those reported in the literature.[18]

### 3,6-Dimethoxy-4,5-methylenedioxy-allylbenzene (apiol)

White powder, m.p. 56°C, C _12_ H _14_ O _4_. MS; *m*/z (%): 222 M^+^ (100); 207 (20); 192(10).

^1^H NMR (300 MHz, CDCl _3_): δ 3.30 (2H, *dd, J* = 1.5; 6.6 Hz, H-7), 3.78 (3H, *s*, OMe-2), 3.94 (3H, *s*, OMe-5), 5.07 (1H, *dd, J* = 1.5; 9.0 Hz, H-9a), 5.13 (1H, *dd, J* = 1.5; 15.4 Hz, H-9b), 5.90 (1H, *m*, H-8), 5.96 (2H, *s*, OCH _2_ O), 6.38 (1H, *s*, H-2).

^13^ C NMR (300 MHz, CDCl _3_): δ 110.8.0 (C-1), 136.5 (C-2), 139.7 (C-3), 135.2 (C-4), 139.2 (C-5), 108.5 (C-6), 34.3 (C-7), 137.5 (C-8), 115.5 (C-9), 60.4 (OMe-2), 57.1 (OMe-5), 101.7 (OCH _2_ O).

### 3-Methoxy-4,5-methylenedioxy-allylbenzene (myristicin)

Colorless oil, C _11_ H _12_ O _3_. MS; *m*/z (%): 192 M^+^(100); 161 (13); 119(16).

^1^H NMR (300 MHz, CDCl _3_): δ 3.25 (2H, d, J = 6.7 Hz, H-7), 3.81 (3H, s, OMe), 5.09 (1H, dd, J = 1.7; 17.1 Hz, H-9a), 5.01 (1H, dd, J = 1.7; 8.0 Hz, H-9b), 5.90 (1H, m, H-8), 5.92 (2H, s, OCH _2_ O), 6.36 (1H, d, J = 1.4 Hz, H-6), 6.39 (1H, d, J = 1.4 Hz, H-2). ^13^C NMR (300 MHz, CDCl _3_): δ 126.0 (C-1), 104.9 (C-2), 137.6 (C-3), 144.5 (C-4), 144.3 (C-5), 102.7 (C-6), 33.8 (C-7), 137.3 (C-8), 115.5 (C-9), 51.0 (OMe-3), 101.0 (OCH _2_ O).

### Antimicrobial activity

The acetone extracts of *A. sicula* aerial parts and the isolated apiol and myristicin were tested *in vitro* for their antimicrobial activity at a screening concentration of 200 μg/mL against the following Gram-positive and Gram-negative human pathogenic reference (ATCC and DSM) bacterial strains: *Staphylococcus aureus*, *Staphylococcus epidermidis*, *Escherichia coli*, and *Pseudomonas aeruginosa*. Antifungal tests were attempted against the reference strains *Candida albicans* and *Candida tropicalis*.

### Antiproliferative activity

All the extracts were also tested for their *in vitro* antiproliferative activity at a screening concentration of 100 μg/mL against K-562, NCI-H460, and MCF-7 cell lines. Antiproliferative effects were estimated in terms of percentage of growth inhibition. Values of growth inhibition of more than 15% at a screening concentration of 100 μg/mL are reported in Table 1.

**Table 1:** Growth inhibition percentage recorded on K-562, NCI-H460, and MCF-7 cell lines at a screening concentration of 100 μg/mL

	K-562	NCI-H460	MCF-7
Leaves extract	90	86.7	65.5
Flowers extract	100	66.2	71.2
Stems extract	95.4	18.2	40.5
Apiol	100	100	100
Myristicin	100	100	100

Values are the mean of at least 3 independent determinations; coefficient of variation was less than 15%

Values of drug concentration at which the cell proliferation was inhibited to 50% of the untreated growth control (IC _50_) were determined when the activity at the screening concentration was higher than 50% [Table 2].

**Table 2:** IC_50_ (μg/mL) recorded on K-562, NCI-H460, and MCF-7 cell lines

	K-562	NCI-H460	MCF-7
Leaves extract	27.5	81	87
Flowers extract	25	73	83
Stems extract	37.5	100	100
Apiol	24.1	43	36
Myristicin	18.5	16	16

Values are the mean of at least 3 independent determinations; coefficient of variation was less than 15%

## DISCUSSION

All acetone extracts, apiol and myristicin, resulted inactive as antimicrobial agents at the maximum tested concentration of 200 μg/mL.

Data reported in Table 2 show significant antiproliferative activity against the 3 cell lines used, in particular against K-562. It was also found that both apiol and myristicin showed cytotoxicity against all the cell lines used. Moreover, this activity was more pronounced for myristicin (IC _50_ values of K-562, NCI-H460, and MCF-7 were 18.5, 16, and 16 μg/mL, respectively).

In present study, the results clearly demonstrate that acetonic extracts of aerial parts of *A. sicula*, apiol and myristicin, induced significant antiproliferative activity on the tested cancer cell lines.

The literature data reported that myristicin has been implicated as responsible for anticancer activities of some medicinal plants; that it has a substantial antiproliferative activity against human cancer and leukemic cells; and it is a cancer chemopreventive agent.[19–23] Other results suggest that apiol induce an efficient suppression of cell proliferation in colon cancer cell lines.[24]

## CONCLUSIONS

Our observations suggest that both apiol and myristicin could be tested as novel treatment in cancer chemotherapy. Further investigations in this direction are required.
